# Investigation of the miRNA and mRNA Coexpression Network and Their Prognostic Value in Hepatocellular Carcinoma

**DOI:** 10.1155/2020/8726567

**Published:** 2020-11-12

**Authors:** Hao Zhang, Xi Chen, Yufeng Yuan

**Affiliations:** Department of Hepatobiliary and Pancreatic Surgery, Zhongnan Hospital of Wuhan University, Donghu Road 169# Wuhan 430071, China

## Abstract

**Purpose:**

To identify pivotal differentially expressed miRNAs and genes and construct their regulatory network in hepatocellular carcinoma.

**Methods:**

mRNA (GSE101728) and microRNA (GSE108724) microarray datasets were obtained from the NCBI Gene Expression Omnibus (GEO) database. Then, we identified the differentially expressed miRNAs and mRNAs. Sequentially, transcription factor enrichment and gene ontology (GO) enrichment analysis for miRNA were performed. Target genes of these differential miRNAs were obtained using packages in *R* language (*R* package *multiMiR*). After that, downregulated miRNAs were matched with target mRNAs which were upregulated, while upregulated miRNAs were paired with downregulated target mRNA using scripts written in Perl. An miRNA-mRNA network was constructed and visualized in Cytoscape software. For miRNAs in the network, survival analysis was performed. And for genes in the network, we did gene ontology (GO) and KEGG pathway enrichment analysis.

**Results:**

A total of 35 miRNAs and 295 mRNAs were involved in the network. These differential genes were enriched in positive regulation of cell-cell adhesion, positive regulation of leukocyte cell-cell adhesion, and so on. Eight differentially expressed miRNAs were found to be associated with the OS of patients with HCC. Among which, miR-425 and miR-324 were upregulated while the other six, including miR-99a, miR-100, miR-125b, miR-145, miR-150, and miR-338, were downregulated.

**Conclusion:**

In conclusion, these results can provide a potential research direction for further studies about the mechanisms of how miRNA affects malignant behavior in hepatocellular carcinoma.

## 1. Introduction

Hepatocellular carcinoma (HCC) is one of the most common cancers in the world. It is estimated that 840,000 new cases of HCC are acquired and at least 780,000 people die of HCC every year, and over half of the global incidence and mortality of HCC occur especially in Eastern Asian [1]. HCC has a high incidence (4.7% of new cancer cases) and the second highest cancer mortality rate (8.2% of cancer-related deaths) worldwide [2], and China accounts for 47% of the total number of HCC cases as well as HCC-related mortality [3]. The development of HCC is closely related to the infection of the hepatitis B virus (HBV) infection, followed by the hepatitis C virus infection (HCV), and related to aflatoxins, alcohol drinking, and so on [4, 5]. In China, HCC has the third highest cancer incidence and has become the second leading cause of cancer-related death, second only to lung cancer [6]. Unfortunately, there are many difficulties in the diagnosis and treatment of HCC, but the most frequent is the lack of methods for early diagnosis as well as the paucity of studies about the molecular mechanisms of tumor initiation and progression. Therefore, we designed the study to explore the molecular mechanisms of HCC carcinogenesis and progression, as well as new relevant molecular markers for early diagnosis.

Recently, noncoding RNA (ncRNA) drawn extensive concern to further illustrate the molecular mechanisms of HCC. MicroRNAs (miRNAs), families of small noncoding RNAs, had been reported that can serve as molecular markers for early diagnosis of a number of tumors [7, 8]. Many of miRNAs (e.g., miR-1247-3p [9] and miR-935 [10]) have been demonstrated to play a role in the progression of HCC by influencing proliferation, invasion, and metastasis of tumor cells as well as other malignant phenotypes. MiRNAs can promote the degradation of mRNAs and inhibit their translation into proteins by binding to the 3′-untranslated region (3′-UTR) of target mRNAs [11]. Thus, studies on miRNAs were important for inquiring the molecular mechanisms of carcinogenesis and to seek novel biomarkers.

Microarray profiling is a kind of high-throughput technique that developed rapidly in recent years, which can be applied to detect differentially expressed miRNAs and genes in cancer and control samples [12]. To find new directions for research in miRNAs and genes, we analyzed miRNA and mRNA microarray datasets to identified differentially expressed miRNAs and genes and explored their potential relationships. Next, we identified the key miRNAs with survival analysis, network analyses, and functional enrichment.

## 2. Materials and Methods

### 2.1. Microarray Data Collection

The raw miRNA and mRNA sequencing data were obtained from the GEO database (https://www.ncbi.nlm.nih.gov/geo/), which represents the largest public repository of microarray data. In this study, one gene expression profile (GSE101728) and one miRNA expression profile (GSE108724) were downloaded from the GEO.

The GSE101728 dataset was composed of seven pairs of HCC and matched adjacent tumor-free tissue sample mRNA expression profiles which were collected during the surgery from HCC patients admitted to the Zhongshan Hospital of Fudan University [13]. The GSE108724 dataset includes the profiling of the miRNA expression in seven pairs of HCC and matched adjacent tumor-free tissues from the same hospital and research team [13]. All the data were obtained in a raw status and normalized with Perl (Perl version 5.32.3) and *R* (version: 4.0.2).

### 2.2. Data Processing

The raw data was downloaded, and the probe ID was transferred to gene symbol or miRNA name. The data from different groups was classified into the normal group and tumor group with packages in *R* language (*R* package *limma*), which was also used to screen the differentially expressed miRNAs and mRNA between the tumor and normal tissues. In the same time, we also calculated the log fold change (logFC), *P* values, and adjusted *P* values (adj. *P* val). In addition, adj. *P* value<0.05 and ∣logFC | >2 were set as the standards of differentially expressed miRNA and mRNA selection. According to the above standards, 37 differentially expressed miRNAs (15 upregulated and 22 downregulated) and 745 differentially expressed mRNAs (30 upregulated and 441 downregulated) were screened.

### 2.3. Prediction of miRNA Target Genes

After extracted the differential miRNAs, packages in *R* language are used to predict the target genes (*R* package *multiMiR*). The packages were published in 2014 [14], but were updated in April 2020 recently. The packages were integration of fourteen databases for prediction of the miRNA target gene, including miRecords (http://c1.accurascience.com/miRecords), miRTarBase (http://mirtarbase.cuhk.edu.cn/php/index.php), TarBase (http://diana.imis.athena-innovation.gr/DianaTools/index.php), and miRDB (http://mirdb.org/). And the intersection of the results of the fourteen databases was taken as the final result of the target gene prediction.

### 2.4. miRNA-mRNA Interaction Network Construction

Investigation of the miRNA and mRNA coexpression network is beneficial for the exploration of the molecular mechanism of hepatocellular carcinoma. To construct the miRNA-mRNA network, including positive and negative relationships between mRNA and miRNA, we extracted 35 miRNAs with target genes from all the 37 differentially expressed miRNAs and 295 target mRNAs from 745 differentially expressed mRNAs. We matched upregulated miRNA with their downregulated target mRNA and downregulated miRNA with their upregulated mRNA. In result, 330 nodes and 481 edges are included in the network. In this study, the miRNA-mRNA network was constructed using a Perl program, followed by visualization using Cytoscape software (version 3.8.0; 64-bit; http://www.cytoscape.org/) [15]. In addition, we obtained a list of 568 oncogenes and 1217 tumor suppressor genes that have been identified based on previous reports. Research on oncogenes has been reported, and the specific list of oncogenes can be downloaded on the OncoGenomics (intOGen) platform (https://www.intogen.org/search) [16]. The list of tumor suppressor genes can be downloaded in the Tumor Suppressor Gene Database (https://bioinfo.uth.edu/TSGene/). We divided the mRNAs in the network into tumor-promoting genes and tumor suppressor genes according to the obtained gene classification data and distinguished by different colors in the network.

### 2.5. Functional and Pathway Enrichment Analysis

We ran a transcription factor enrichment analysis and a GO enrichment analysis for differential miRNA for the three GO domains: molecular functions (MF), biological processes (BP), and cellular components (CC). A KEGG pathway analysis and a GO enrichment analysis, including MF, BP, and CC, of the mRNAs were subsequently performed. All the enrichment analysis was performed using FunRich software (version 3.1.3; http://www.funrich.org). *P* < 0.05 was applied as the criterion.

### 2.6. Overall Survival (OS) Analysis of the miRNAs

The related clinical data of HCC patients were downloaded from The Cancer Genome Atlas (TCGA, https://portal.gdc.cancer.gov/) database. A total of 368 HCC patients with complete available miRNA expression and follow-up datasets were involved in the overall survival analysis. Patients were divided into two groups according to the median value of the expression of miRNA that we want to study (high vs. low expression). Kaplan-Meier survival analysis was performed, and *P* value of 5-year survival rates was calculated and displayed. For all the analysis and plotting process, the *R* package *Survival* (https://cran.r-project.org/web/packages/survival/index.html) was used.

### 2.7. Analyzed the Expression Data Downloaded from the TCGA Database

In addition to relevant clinical data, we have also downloaded data on the miRNA expression in normal tissues and liver cancer tissues in TCGA. The aforementioned methods are used to process the data, to screen out the miRNAs that are abnormally expressed in liver cancer tissues, and to perform relevant multifactor COX regression analysis and survival analysis for differential miRNAs. Based on miRNAs that have an impact on the prognosis, target genes are predicted, and an interactive network of miRNA and mRNA is constructed. For oncogenes and tumor suppressor genes in the network, we also conducted survival analysis and calculated the difference in five-year survival rates.

## 3. Results

**Figure 1:**
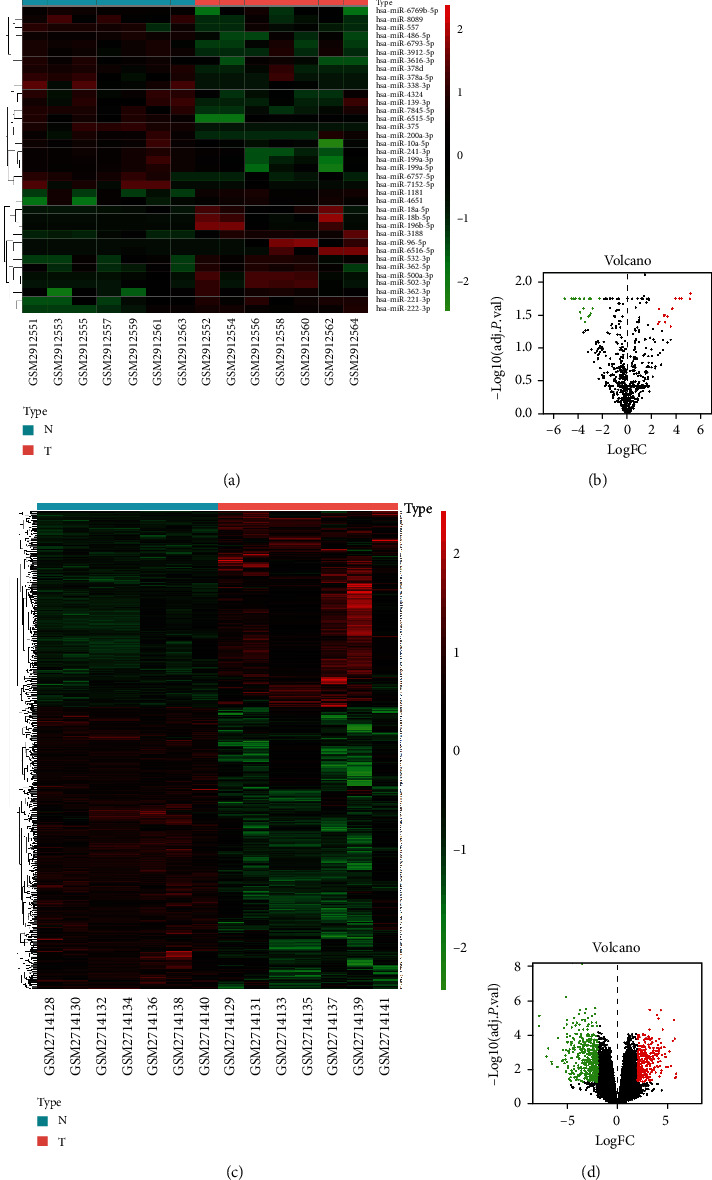
Heatmap and volcano plot of differentially expressed miRNAs and mRNAs. (a) Heatmap of differential miRNA microarray. (b) Volcano plot of differential miRNA microarray. (c) Heatmap of differential mRNA microarray. (d) Volcano plot of differential mRNA microarray.

A total of 745 differentially expressed genes were identified from GSE101728 and 37 miRNAs from GSE108724. The conditions of judgment for significant differences are the logfoldchange > 2 and the adjusted *P* value <0.05. There are 304 upregulated genes and 441 downregulated genes among these differential genes. For miRNA, 15 miRNAs are upregulated, and 22 are downregulated (Figure 1). Heatmap of miRNA and mRNA differentially expression clearly distinguished HCC tissues (posterior seven samples) from paired adjacent normal tissues (Figures 1(a) and 1(c)). In the heatmap, red represents high expression, and green represents low expression. The comparison of colors can show the difference in the expression of miRNA and mRNA in the two sets of samples. In the volcano plot, green and red dots indicated the down and upregulation of the miRNA and mRNA expression in tumor and normal tissues, respectively. In the volcano map of miRNA, you can see the expression distribution of miRNA. Since the fold change is set to 4 times, most miRNAs are considered to have no expression differences, and only a small part of miRNAs are included in subsequent studies (Figures 1(b) and 1(d)).

**Figure 2:**
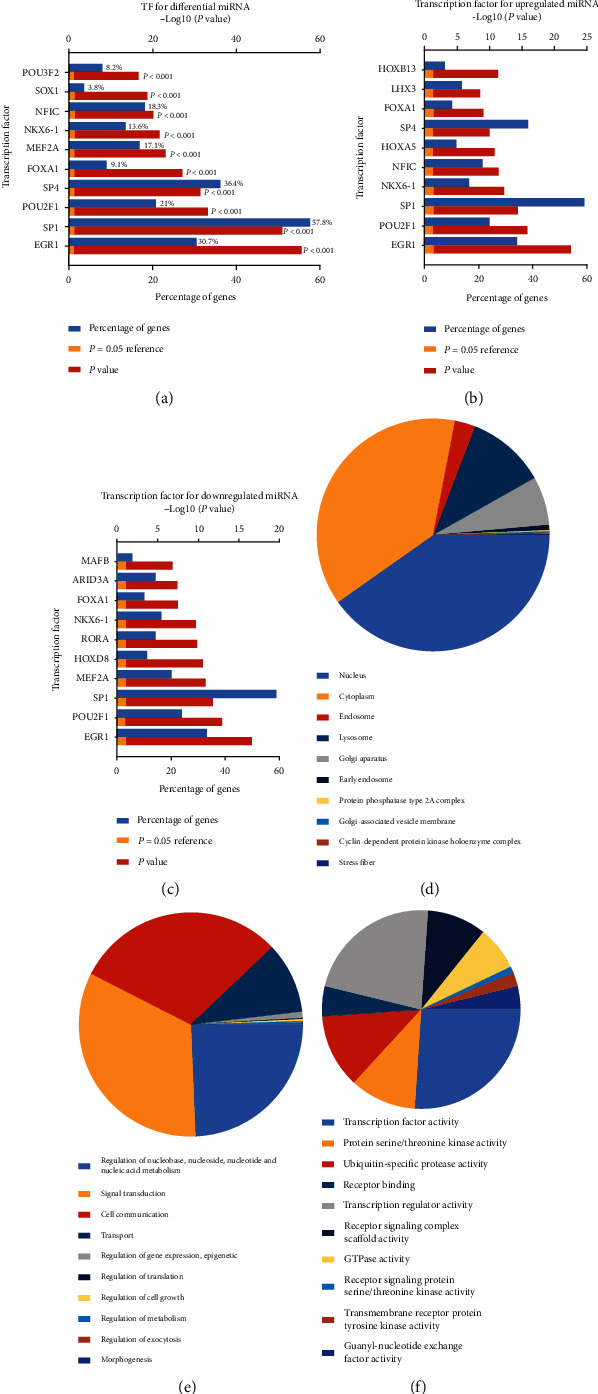
Enrichment analysis of transcription factors and GO terms for differential miRNA. (a) Transcription factor (TF) enrichment analysis for differential miRNA. (b) TF enrichment analysis for upregulated miRNA. (c) TF enrichment analysis for downregulated miRNA. (d) The enriched GO terms in the cellular component (CC). (e) The enriched GO terms in the biological process (BP). (f) The enriched GO terms in the molecular function (MF).

For the selected differential miRNAs, we performed GO and transcription factors (TF) enrichment analysis. TF and GO analysis are hints for the research direction. Through the GO analysis, we can find the GO classification items of the differential miRNAs and find out which function changes may be related to the differential miRNAs. The results of TF and GO enrichment analysis of the differential miRNAs are shown in Figure 2. The results of TF analysis for differential upregulated and downregulated miRNA are shown below (Figure 2(a)–(c)). In the result of transcription factor enrichment, the blue column indicates the proportion of miRNA enriched on the transcription factor to all differential miRNAs. The red bar represents the value of -log_10_ (*P* value), and the yellow bar represents the threshold of statistical difference (-log_10_ 0.05, *P* = 0.05). The red bar is longer than the yellow band, indicating a statistical difference (*P* < 0.05). GO analyses cover three domains and show the top 10 significant GO enrichments according to enrichment scores [-log_10_ (*P* value)] (Figures 2(d)–(f)).

**Figure 3:**
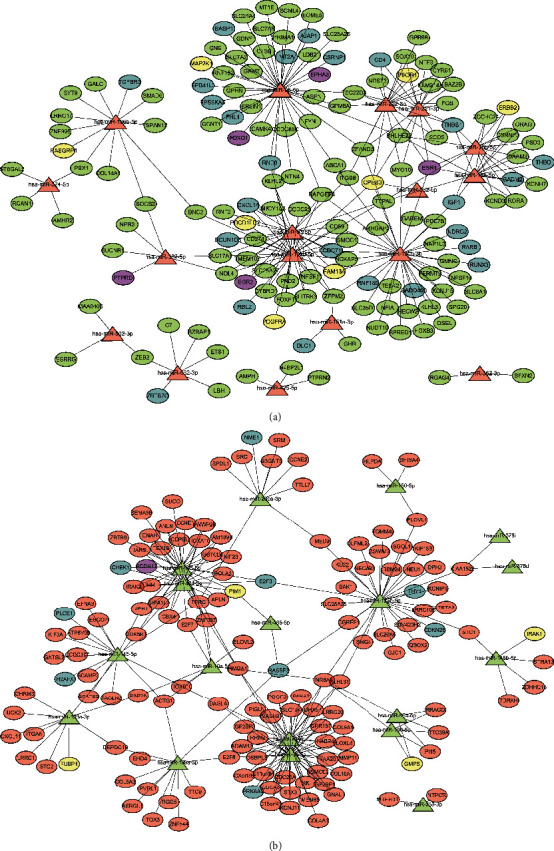
The miRNA-mRNA regulatory network consists of 35 miRNAs and 295 target mRNAs. (a) Regulatory network of upregulated miRNAs and downregulated mRNAs. (b) Regulatory network of downregulated miRNAs and upregulated mRNAs. Triangular nodes, miRNA; elliptical nodes, mRNA; green nodes, downregulation; pink nodes, upregulation; blue nodes, tumor suppressor gene; yellow nodes, oncogenes; purple nodes, at the same time in the list of oncogenes and tumor suppressor genes.

In order to show the relationship between differential miRNAs and target genes more intuitively, we used Cytoscape software to achieve visualization. The miRNA-mRNA regulatory network was constructed that consists of 35 miRNAs and 295 target mRNAs. Since miRNAs generally negatively regulate target genes, 17 upregulated miRNAs were matched with 144 downregulated target mRNAs while 18 downregulated miRNAs with 151 target mRNAs (Figure 3). In the network, we use different colors to represent different expression levels and different functions of miRNA and mRNA.

**Figure 4:**
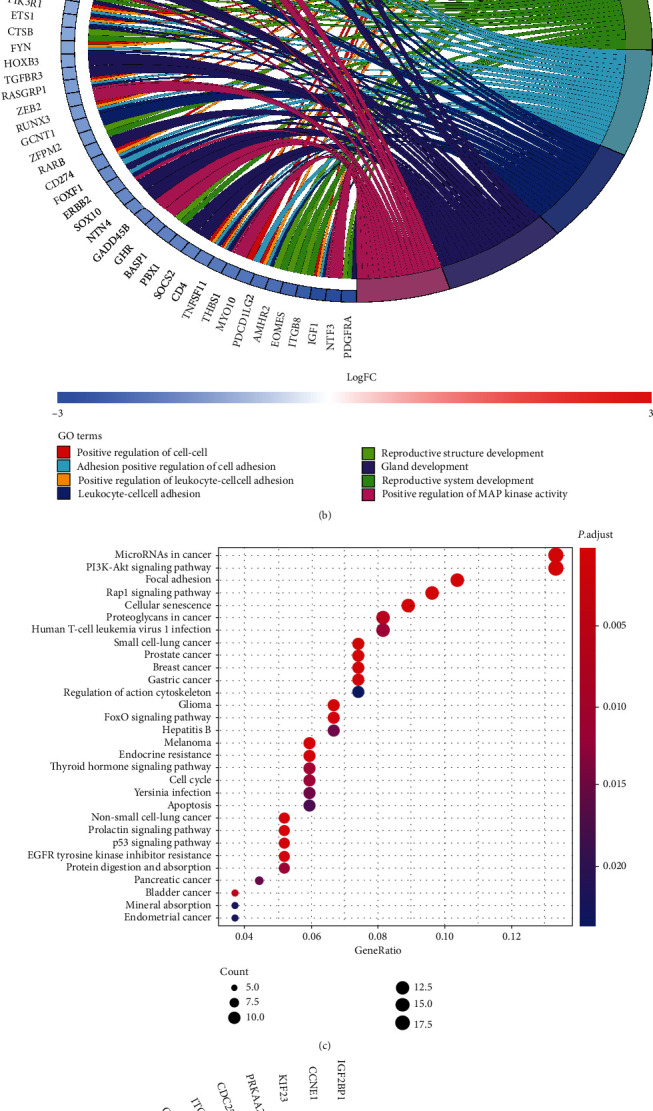
Bubble plot and circle plot of the GO/KEGG analysis of differentially expressed genes in the miRNA-mRNA network. (a) Bubble plot of the GO enrichment analysis. (b) Circle plot of the GO enrichment analysis. (c) Bubble plot of the KEGG enrichment analysis. (d) Circle plot of the KEGG enrichment analysis.

We not only performed GO enrichment analysis for miRNAs but also GO and KEGG enrichment analysis for differential mRNAs. GO enrichment analysis and KEGG pathway analysis of differentially expressed genes in the miRNA-mRNA network are shown in Figure 4. Representation of the genes in the GO enrichment bubble plot and circle plot displayed the count distribution in BP, CC, and MF. Bubble color intensity indicates fold enrichment of GO terms overrepresented in that cluster of genes, and the size corresponds to the number of genes enriched (count). The GO analysis results revealed that the differentially expressed genes were significantly enriched in the terms “reproductive structure development,” “reproductive system development,” “positive regulation of cell adhesion,” etc (Figures 4(a) and 4(b)). The result of the KEGG pathway analysis was shown in the same form. The genes were significantly enriched in “MicroRNAs in cancer,” “PI3K-Akt signaling pathway,” and “Focal adhesion.” The results of the enrichment analysis suggest that the effect of differential genes enriched in the corresponding items on the corresponding phenotype of tumor cells can be studied. For example, the KEGG enrichment analysis results of SRC show that it is enriched in the item of cellular adhesion, so basic experiments can be used to verify whether SRC has an effect on the invasion and metastasis of liver cancer cells..

**Figure 5:**
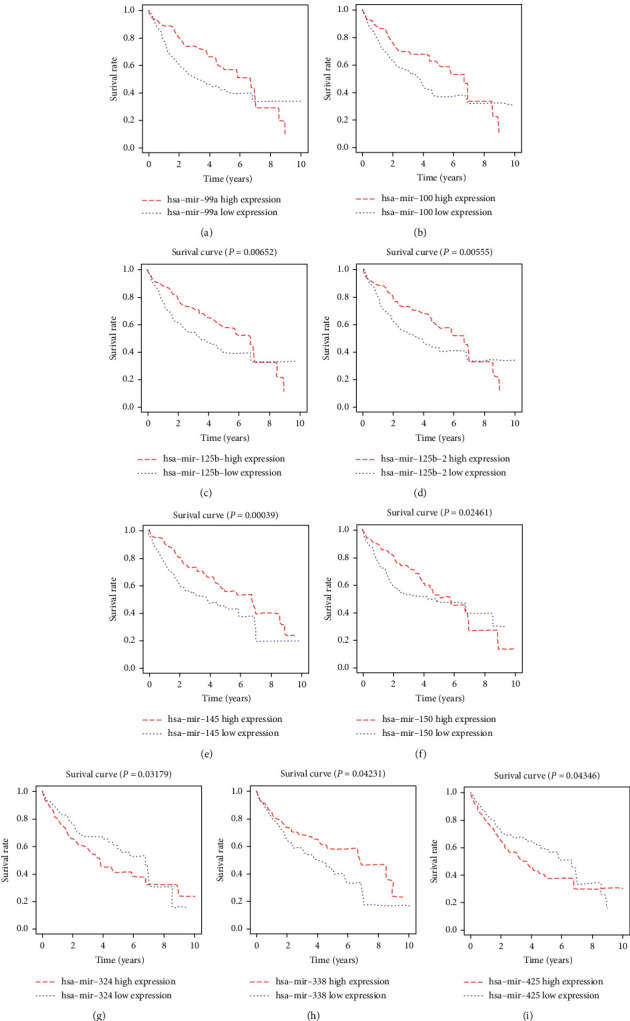
Overall survival analysis of high and low expression groups of (a) miR-99a, (b) miR-100, (c) miR-125b-1, (d) miR-125b-2, (e) miR-145, (f) miR-150, (g) miR-324, (h) miR-338, and (i) miR-425 in HCC.

If the difference in the expression of miRNAs is significantly related to the survival of patients, it means that this miRNA is likely to have greater research value. Therefore, for differential miRNAs in the network, OS analysis was performed. The data including the expression of miRNAs, follow-up time, survival state, and survival time were from the TCGA miRNA-seq dataset. Survival curves were plotted, and differences between survival curves were estimated (Figure 5). As a result, nine survival curves are statistically different (*P* <0.05); among them, hsa-mir-125b was divided into hsa-mir-125b-1 and hsa-mir-125b-2 (Figures 5(c) and 5(d)).

**Figure 6:**
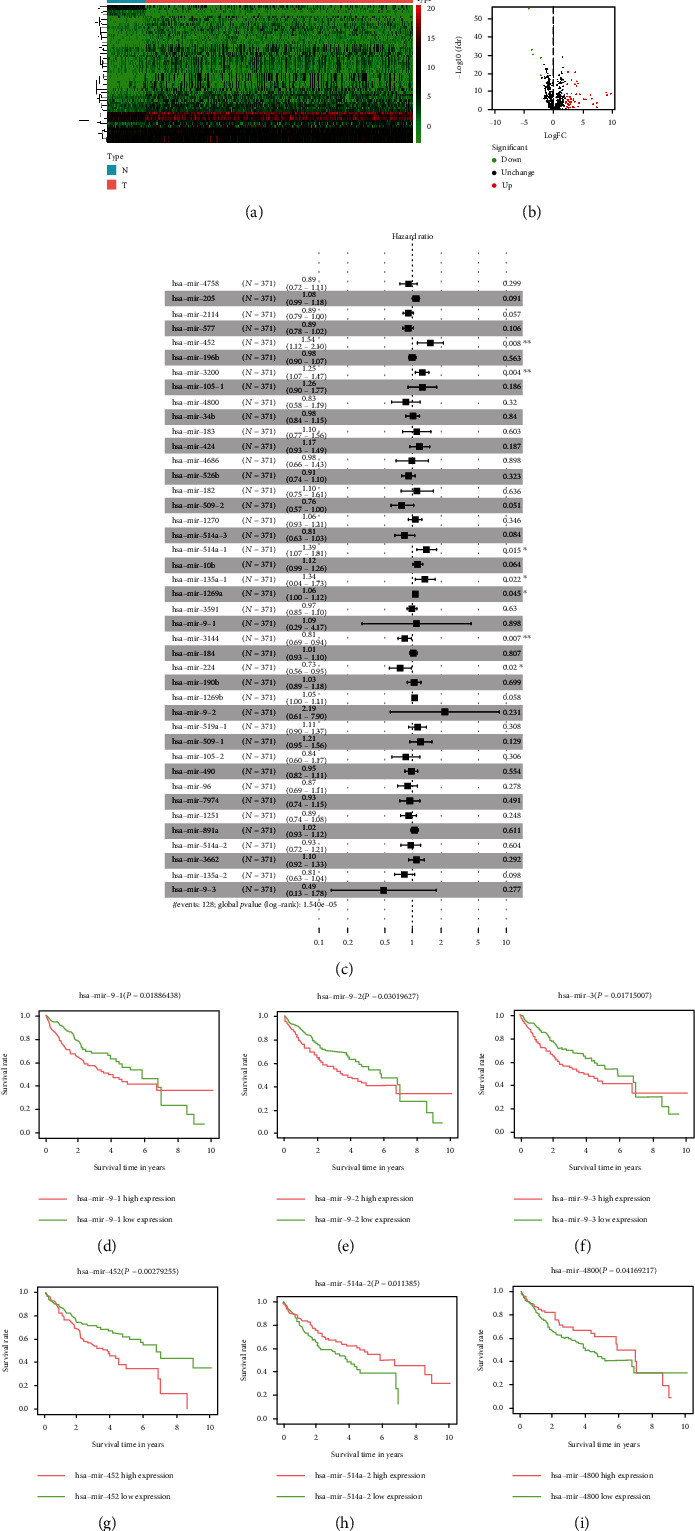
Analysis results of differential miRNA in TCGA. (a) Heatmap of differential miRNAs. (b) Volcano plot. (c) Multivariate COX regression analysis result. (d) Survival analysis curve of miR-9-1. (e) Survival analysis curve of miR-9-2. (f) Survival analysis curve of miR-9-3. (g) Survival analysis curve of miR-452. (h) Survival analysis curve of miR-514a-2. (i) Survival analysis curve of miR-4800.

In addition to the GEO database, we also conducted data mining on the TCGA database. Perform data processing on the data downloaded from the TCGA database screen out a total of 53 abnormally expressed miRNAs and calculate the *P* value. All the different miRNAs are displayed in the chart and sorted by *P* value (Table 1). We drew a heatmap and a volcano map to visually show the difference in the expression and distribution of 53 differential miRNAs in normal tissues and cancer tissues (Figures 6(a) and 6(b)). In order to study the impact of differential miRNAs on the prognosis of patients, we used multivariate COX regression analysis and survival analysis methods. Multivariate COX regression analysis showed that 8 miRNAs are related to the prognosis of patients (*P* < 0.05). At the same time, we also calculated the hazard ratio of each miRNA. A hazard ratio greater than 1 indicates a negative impact on the prognosis, and the hazard ratio that is less than 1 indicates a benign effect on the prognosis (Figure 6(c)). Then, we drew survival curves for all the differential miRNAs based on the expression, the patient's survival time, and survival status and calculated the 5-year survival rate. The results showed that the 5-year survival rates of mir-9-1, mir-9-2, mir-9-3, mir-452, mir-514a-2, and mir-4800 were significantly different (Figures 6(d)–(i)).

**Figure 7:**
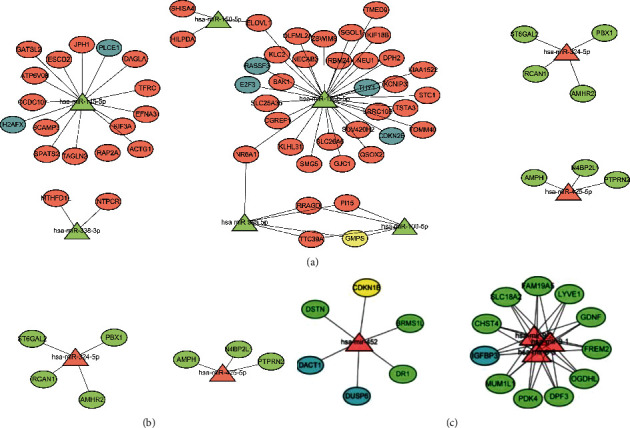
The miRNA-mRNA regulatory network consists of miRNA with OS analysis difference and their target mRNA. (a) Network of downregulated miRNAs with OS analysis difference and their target mRNAs. (b) Network of upregulated miRNAs with OS analysis difference and their target mRNAs. (c) Network of upregulated miRNAs in the TCGA database with OS analysis difference and their target mRNAs in the legend. Triangular nodes, miRNAs; elliptical nodes, mRNAs; green nodes, downregulation; pink nodes, upregulation; blue nodes, tumor suppressor gene; yellow nodes, oncogenes.

To find more meaningful target-regulatory relationships and guide the next experiments, we constructed a network which only contains miRNAs with significant differences in the survival curves (Figure 7). In the network, we also marked the oncogenes and tumor suppressor genes with different colors.

**Figure 8:**
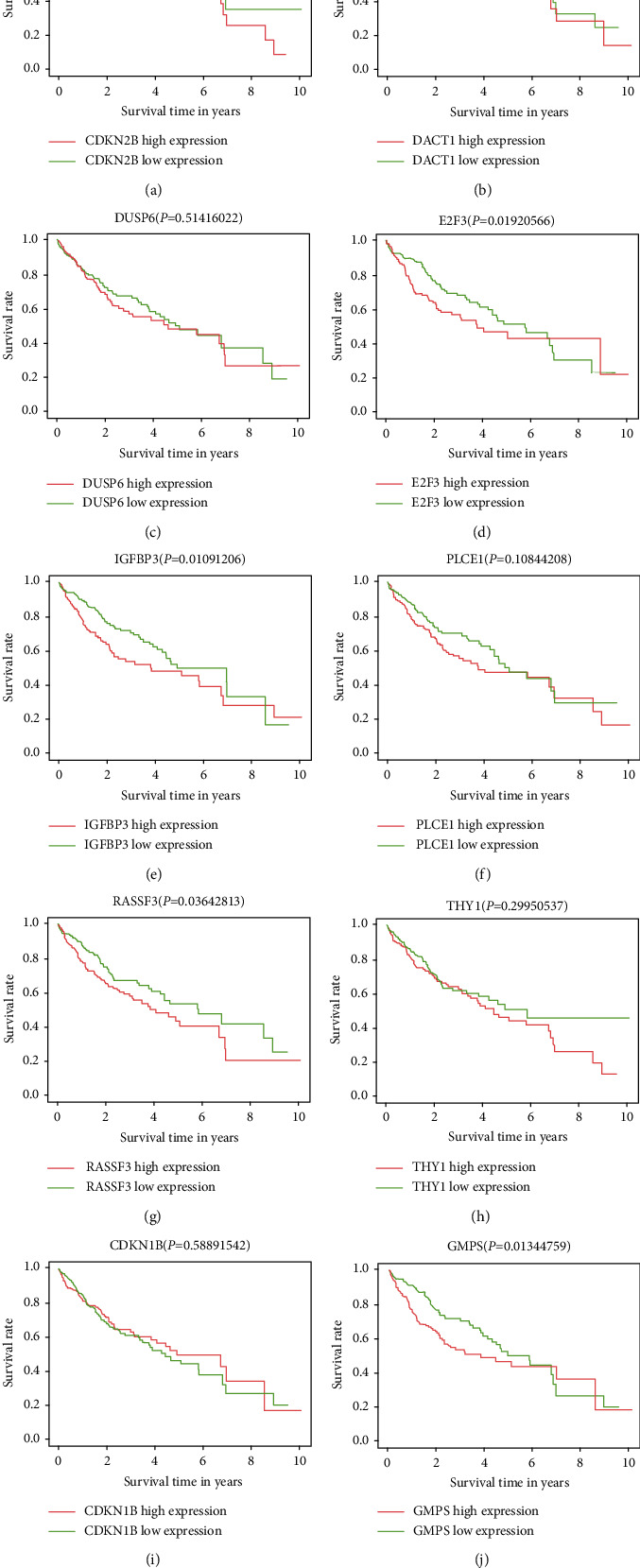
Survival analysis curves of oncogenes and tumor suppressor genes in the network. (a) CDKN2B. (b) DACT1. (c) DUSP6. (d) E2F3. (e) IGFBP3. (f) PLCE1. (g) RASSF3. (h) THY1. (i) CDKN1B. (j) GMP3.

For oncogenes and tumor suppressor genes in the network, we conducted survival analysis and calculated the difference in five-year survival rates (Figure 8). We conducted survival analysis on the six tumor suppressor genes CDKN2B, DACT1, DUSP6, E2F3, IGFBP3, PLCE1, RASSF3, and THY1 and found that E2F3, IGFBP3, and RASSF3 genes had a significant impact on the 5-year survival rate of patients (*P* < 0.05). The 5-year survival rate of the oncogene GMPS high expression group was significantly lower than that of the low expression group.

## 4. Discussion

With a high mortality rate, substantial morbidity, and the increasing trend of the incidence rate of HCC worldwide, the pathogenesis, disease progression, and treatment of HCC are worthy of further study and exploration. Noncoding RNA has been an intensive research topic in molecular biology for several years and the focus of numerous studies [17, 18]. MiRNAs, families of small noncoding RNAs, played important roles in nearly all biological processes. Plenty of studies indicated that the abnormal expression of miRNAs may contribute to oncogenesis and the progression of HCC by inhibiting target genes through the degradation of their target mRNAs or by inhibiting translation [19, 20]. Thanks to the well-developed microarray technology, now it is easier to determine the expression levels of the miRNAs and mRNAs. We can identify differentially expressed miRNAs and genes between normal and tumor tissues and screen miRNAs that seem to play roles in tumor onset or progression. In addition, the result of microarray analysis can help give direction for future research.

In the study, a total of 35 miRNAs (17 upregulated and 18 downregulated) and 295 mRNAs (151 upregulated and 144 downregulated) were screened. The transcription factors for differentially expressed miRNA were enriched in EGR1, POU2F1, SP1, MEF2A, HOXD8, etc. GO enrichment analysis of these miRNAs showed that they are significantly enriched in “regulation of nucleobase, nucleoside, nucleotide and nucleic acid metabolism”, signal transduction, cell communication, and transport. The differentially expressed mRNA is enriched in the reproductive structure development, reproductive system development, and positive regulation of cell adhesion. For KEGG analysis of mRNAs, they are enriched in miRNAs in cancer, PI3K-Akt signaling pathway, focal adhesion, and Rap1 signaling pathway. Moreover, by constructing the miRNA-mRNA network and performing OS analysis, we identified miRNAs including miR-99a, miR-100, miR-125b, miR-145, miR-150, miR-324, miR-338, and miR-425, which were found to have an impact on the HCC survival rate. Thus, the network has been simplified.

miR-125b-5p is one of the downregulated miRNAs in tumor tissues of HCC patients compared to normal tissues. In the miRNA-mRNA network, it has the highest connectivity with target genes, which regulates 31 upregulated genes. Among these, there are 2 genes in the top 100 of total 979 upregulated genes including KIF18B and RBM24. Little research has been done on miR-125b in HCC, but some studies showed that miR-125b can affect the metastasis of gastric cancer cells and inhibit colorectal cancer proliferation [21, 22]. Recent research reported that KIF18B promotes hepatocellular carcinoma progression through activating the Wnt/*β*-catenin-signaling pathway [23], but the upstream regulators of KIF18B are unknown. According to the network, the exact relationship between miR-125b and KIF18B needs to be verified through experiments.

Among target genes of miR-99a, peptidase inhibitor 15 (PI15) has been reported to act as a novel blood diagnostic marker for cholangiocarcinoma, and the plasma PI15 level in HCC patients was clearly higher than normal [24]. However, the physiological and pathological role of plasma PI15 is still unknown. Downregulation of GMP synthetases (GMPS), another target gene of miR-99a, can result in reduced cell viability as a p53 repression target in HCC [25]. Thus, the downregulation of miR-99a may inhibit tumor proliferation by upregulating GMPS, but it still requires experimental validation. Recent research showed that nuclear receptor subfamily 6, group A, member 1 (NR6A1) regulates lipid metabolism of HepG2 cells, and the positive expression of NR6A1 is a novel marker of disease progression and aggressiveness in prostate cancer patients [26, 27]. Thus, the interaction between miR-99a and NR6A1 in tumor migration and invasion could be a direction for future research. In addition, a ceRNA network including miR-125b and miR-99a could also be constructed.

For upregulated miRNA, miR-324-5P is correlated to patients' prognosis and has regulatory relationships with 4 genes. One research showed that miR-324-5p suppresses HCC cell invasion, but another study reported that lncRNA-85 promotes HCC cellular proliferation and migration by targeted binding and regulating miR-324-5p [28, 29]. Thus, the effect of miR-324-5P on tumor progression might be different via different mechanisms. Among target genes, the high expression of alpha-2,6-sialyltransferase 2 (ST6GAL2), one of the top 100 of all the 1236 downregulated genes, was demonstrated to promote tumorigenesis of follicular thyroid cancer via activating the Hippo signaling pathway [30], and the downregulation of ST6GAL2 is associated with improved patient survival in breast cancer [31], but the effects of ST6GAL2 have not been reported yet on the oncogenesis and the progression of HCC. Regulator of calcineurin 1(RCAN1) is broadly expressed in the liver, placenta, and other tissues. Overexpressed RCAN1, as a potential target of miR-572, induced apoptosis of HCC cells and inhibited cell proliferation and invasion [32]. The regulatory relationship between miR-324-5P and RCAN1 has not been reported. Anti-Mullerian hormone receptor type 2 (AMHR2) encodes the receptor for the anti-Mullerian hormone (AMH) which results in male sex differentiation. PBX1 encodes a nuclear protein that belongs to the PBX homeobox family of transcriptional factors. The former two genes have a greater value of study than the latter.

MiR-425-5p promotes tumor progression in HCC [33], gastric cancer [34], breast cancer [35] and so on. Amphiphysin (AMPH) is a critical tumor suppressor that inhibits tumor progression in breast cancer [36], osteosarcoma [37], etc. Thus, the upregulation of miR-425 may negatively regulate AMPH to promote tumor progression. The relationship between miR-425 and AMPH has been reported that miR-425 regulates cell proliferation, migration, and apoptosis by targeting AMPH in non-small-cell lung cancer [38]. However, the relationship needs to be validated in HCC in further studies. There are few reports about the roles and mechanisms of NEDD 4 binding protein 2-like 1 (N4BP2L1) and protein tyrosine phosphatase receptor type N2 (PTPRN2) for their limited value.

Some miRNAs have tumor suppressor and carcinogenic effects, and the main mechanism is the binding of miRNA and target mRNA. The combination of miRNA and mRNA will cause a decrease in the expression level of target mRNA. Some studies have also found that miRNAs can bind to target mRNA to increase the translation of target mRNA [39]. Through these mechanisms, miRNAs can regulate the expression of many genes and play a similar role to oncogenes or tumor suppressor genes. In the interaction network, we have also marked out the oncogenes and tumor suppressor genes that have been identified. miRNAs and mRNAs transcribed from these genes have potential interaction and coexpression relationships. This can provide certain research directions for future research.

We identified 37 differentially expressed miRNAs and 745 mRNAs in tumor tissues of HCC patients compared to normal controls. 481 negatively regulatory pairs were used to construct a miRNA-mRNA interaction network including 35 miRNAs and 295 mRNAs. Then, we identified 8 miRNAs that are associated with the long-term survival rate and prognosis by using survival analysis. GO and KEGG pathway analyses revealed that the abnormal expression of miRNAs and genes may participate in the regulation of cell adhesion and then induce invasion and metastasis of tumor cells. There are limitations to the study. The sample size is relatively small, which may have an impact on the trustworthiness and credibility of the result of microarray analysis. In a further study, we can get a large sample size of differentially expressed genes in miRNA or mRNA microarray datasets for screening differential miRNAs and mRNAs. Furthermore, the mechanisms of miRNA–mRNA regulatory relationship in the network require validation through laboratory-based experiments.

## Figures and Tables

**Table 1:** Differentially expressed miRNAs in HCC in the TCGA database.

ID	LogFC	*P* value	ID	LogFC	*P* value
hsa-mir-4686	-4.1834	4.12E-59	hsa-mir-1251	5.1093	3.16E-07
hsa-mir-490	-3.6287	2.93E-36	hsa-mir-1269a	5.4367	4.14E-07
hsa-mir-1258	-3.4329	1.10E-33	hsa-mir-6783	2.3317	7.11E-07
hsa-mir-424	-2.1563	1.88E-31	hsa-mir-514a-3	2.5705	7.32E-07
hsa-mir-4746	2.4126	2.70E-23	hsa-mir-9-3	3.3295	8.76E-07
hsa-mir-10b	3.6625	4.32E-23	hsa-mir-9-2	3.3379	1.35E-06
hsa-mir-4800	-2.1605	3.04E-21	hsa-mir-7974	2.6364	6.24E-06
hsa-mir-224	2.9886	2.26E-18	hsa-mir-135a-2	3.5473	6.50E-06
hsa-mir-34c	3.9009	1.19E-17	hsa-mir-4758	2.1997	9.23E-06
hsa-mir-183	4.0866	2.11E-16	hsa-mir-135a-1	3.0362	1.09E-05
hsa-mir-452	2.3124	4.58E-16	hsa-mir-1270	2.0524	1.32E-05
sa-mir-182	3.5976	5.59E-16	hsa-mir-509-2	2.8006	2.43E-05
hsa-mir-96	3.9076	5.07E-15	hsa-mir-520a	7.3327	3.57E-05
hsa-mir-767	8.8785	4.72E-11	hsa-mir-184	4.3001	4.22E-05
hsa-mir-190b	2.9875	1.48E-10	hsa-mir-3591	2.0458	6.92E-05
hsa-mir-105-2	9.6441	1.75E-10	hsa-mir-514a-2	2.4440	0.00012
hsa-mir-3200	2.3078	1.89E-10	hsa-mir-526b	6.6936	0.00020
hsa-mir-34b	3.8027	4.14E-10	hsa-mir-509-3	2.8453	0.0002
hsa-mir-3144	4.4032	5.12E-10	hsa-mir-509-1	2.5959	0.0006
hsa-mir-891a	6.2658	1.04E-09	hsa-mir-205	4.1754	0.00467
hsa-mir-105-1	8.9992	1.32E-09	hsa-mir-1224	2.3070	0.00717
hsa-mir-3662	2.7909	1.03E-08	hsa-mir-2114	3.2124	0.00741
hsa-mir-508	2.2759	1.03E-08	hsa-mir-519a-1	7.3346	0.01289
hsa-mir-1254-1	2.6607	1.33E-08	hsa-mir-196b	3.4244	0.01445
hsa-mir-1269b	6.4907	1.62E-07	hsa-mir-541	2.7758	0.01715
hsa-mir-514a-1	2.9022	2.37E-07	hsa-mir-577	2.5328	0.02511
hsa-mir-9-1	3.3532	2.92E-07			

## Data Availability

The mRNA (GSE101728) and microRNA (GSE108724) microarray datasets during the current study are available in the Gene Expression Omnibus database (https://www.ncbi.nlm.nih.gov/geo/).

## Additional Points

In this paper, we analyzed miRNA and mRNA microarray datasets to identified differentially expressed miRNAs and genes and explored their potential relationships. And we identified the key miRNAs with survival analysis, network analyses, and functional enrichment. This study can provide a research direction for further study of molecular mechanism. We believe this manuscript is valuable for all the researchers who are interested in. This study includes not only the research about tumor-related miRNAs and genes but also the clinical research about differential miRNAs.
